# Estimating Factors Related to Fluoroquinolone Resistance Based on One Health Perspective: Static and Dynamic Panel Data Analyses From Europe

**DOI:** 10.3389/fphar.2019.01145

**Published:** 2019-10-03

**Authors:** Dandan Zhang, Youwen Cui, Xinping Zhang

**Affiliations:** School of Medicine and Health Management, Tongji Medical College, Huazhong University of Science and Technology, Wuhan, China

**Keywords:** fluoroquinolone resistance, One Health, antimicrobial consumption, medical staff, veterinarians, panel data, Europe

## Abstract

**Objectives:** Antimicrobial resistance (AMR) has become **a One** Health problem in which fluoroquinolone resistance has caused great concern. The aim of this study is to estimate factors related to fluoroquinolone resistance involving the professionals and antimicrobial consumption (AMC) in human and animal fields.

**Methods:** A country-level panel data set in Europe from 2005 to 2016 was constructed. The dependent variables were measured by ***Escherichia coli*** (***E. coli***) and ***Pseudomonasaeruginosa*** (***P. aeruginosa***) resistance rates to fluoroquinolones. Both the static and dynamic panel data models were employed to estimate the above factors associated with the resistance rates.

**Results:** The 10% increase in the number of medical staff and veterinary professionals per 100,000 population were significantly correlated with the 32.44% decrease of ***P. aeruginosa*** and 0.57% decrease of ***E. coli*** resistance rates to fluoroquinolones (Coef. = −3.244, −0.057; p = 0.000, 0.030, respectively). The 10% increase in the human AMC was correlated with 10.06% and 8.04% increase of ***P. aeruginosa*** resistance rates to fluoroquinolones in static and dynamic models (Coef. = 1.006, 0.804; p = 0.006, 0.001, respectively). The 10% increase in veterinary AMC was related to a 1.65% decrease of ***P. aeruginosa*** resistance rates to fluoroquinolones (Coef. = −0.165, p = 0.019).

**Conclusions:** The increases in medical and veterinary professionals are respectively associated with the decrease of *P. aeruginosa* and *E. coli* resistance rates to fluoroquinolones. The increase in human AMC is also associated with increase of *P. aeruginosa* resistance rates, while the increase in veterinary AMC was found to be associated with a decrease in resistance rate for *P. aeruginosa*.

## Introduction

Antimicrobial resistance (AMR) has become a global health crisis. Deaths attributed to drug-resistant infections will surpass 10 million in 2050, resulting in an estimated $US100 trillion loss in global economic output if the rising trend is not properly contained from the current level of 700,000 deaths annually (Muller et al., 2003; O’Neill, 2016). Widespread antimicrobial consumption (AMC) in humans and animals is considered to be the major trigger for the severity of AMR. Additionally, there are other main drivers promoting the spread of resistant bacteria and their genes locally and globally, such as severe hospital infection, environmental contamination, and geographical movement of infected humans and animals (Holmes et al., 2016). Antibiotic-resistant bacteria and genes could move relatively easily within and between different reservoirs. Therefore, it is necessary to address the resistance problem by taking the complexity and ecological nature into account from a comprehensive, multi-sectoral perspective, such as One Health (Kahn, 2017).

The concept of “One Health,” introduced at the beginning of the 2000s, was defined and highly emphasized as a collaborative and trans-disciplinary approach of multiple disciplines working together to achieve optimal health for people, animals, and environment (Robinson et al., 2016). The 71st session of the United Nations General Assembly identified AMR as a dominant global health concern, encouraging national policy makers, international organizations, and financial institutions in developed and developing countries to take action towards reducing AMC in both human medicine and agriculture as soon as possible. In general, the initiatives of combating AMR based on the One Health perspective revolve around guiding principles of improving the awareness of antimicrobial use and consequences, reducing the incidence of AMR, encouraging developing new antimicrobials, and taking measures to control AMC in farming and livestock (Yang and Buttery, 2018).

Although many studies have explored AMR and its related factors, most of them use cross-sectional regression in specific settings or countries without considering the emergence of AMR as a continuous dynamic process in practice. This could perhaps be an indication that the effect of influencing factors on AMR may well require panel data analysis. Panel studies are fast displacing their cross-sectional counterpart in sociological research (Halaby, 2004). For research aimed at variation across large-scale social units, panel data proliferate on subjects ranging from welfare spending and poverty (Huber and Stephens, 2000; Moller et al., 2003) to political violence (Villarreal, 2002). In recent years, quantitative studies explored the issue of AMR by constructing panel data analysis, which is acknowledged for its ability to mitigate unobserved heterogeneity. Evidence from studies analyzing AMR by macro country-level thinking was gradually enriched. Within this context, Liu et al. employed a provincial-level panel data set from 2009 to 2016 to estimate the relationship between medical staffing and antimicrobial stewardship performance in China (Liu et al., 2018). Cui et al. used annual national-level penal data from the EU to establish the relationship between AMR and laboratory capabilities by conventional static panel data analysis (Cui et al., 2019).

Based on this existing research, we employed static and dynamic models in panel studies to explore factors related to the resistance of fluoroquinolones (FQs), which are regarded as a critically important antimicrobial for human medicine and one of the antibiotic groups most frequently administered in veterinary medicine (Van et al., 2019). Consequently, the emergence of resistance to FQs has also become a major public health concern (Almalki et al., 2017). The study is expected to produce new evidence or knowledge to understand the relationship between FQ resistance and its influencing factors in the One Health perspective.

## Materials and Methods

### Study Design and Data Sources

We created an annual national-level panel data set on AMR and its factors in human and animal fields covering 29 countries in the EU from 2005 to 2016, due to the data availability. Description of data availability and included countries are exhibited in **Supplementary Table 1**. The variables in the data set included: the AMR rates, the number of medical staff and veterinary professionals per 100,000 population, and AMC in humans and animals.

The AMR rates were collected from the European Antimicrobial Resistance Surveillance Network (EARS-Net), which included *Escherichia coli* (*E. coli*) and *Pseudomonas aeruginosa* (*P. Aeruginosa*) resistance rates to FQs. They were selected as the dependent variables for the following reasons: *E. coli* is a ubiquitous enteric commensal in both human and veterinary species. *P. aeruginosa*, an important causative agent of food infection, also has high mortality and morbidity rates compared with other pathogens of healthcare-associated infections (Driscoll et al., 2007). Moreover, increased use of FQs for the treatment of infections caused by Enterobacteriaceae and *P. aeruginosa* has led to an increase in the resistance rates over time (Dalhoff, 2012).

The numbers of medical staff including physicians, pharmacists, and nurses in practice were extracted from the Eurostat Database. The numbers of veterinary professionals were originated from database of World Organization for Animal Health (OIE). The numbers of professionals in human and veterinary fields were then converted into the numbers per 100,000 population by dividing the total annual population of each country from the Eurostat Database.

The AMC in humans was originated from the European Surveillance of Antimicrobial Consumption Net (ESAC-Net). The AMC in humans was the sum of AMC from the community (primary care) and hospitals. The community AMC, which represents around 90% of the total AMC, was used as a surrogate for the total consumption when hospitals’ AMCs were not available (European Centre for Disease Prevention and Control et al., 2015). Human AMC was reported in defined daily doses (DDDs) per 1,000 inhabitants and per day. The ESAC-Net data set allowed recalculation of the weight of the antimicrobial at the substance level based on the numbers of DDD. According to the Anatomical Therapeutic Chemical (ATC) classification and the ATC/DDD index defined by the World Health Organization (WHO) Collaborating Centre for Drug Statistics Methodology (WHO CC), every substance of a specific ATC code had its own weight. The weight sums were expressed in mg/kg human biomass by using the standard human body weight 62.5 kg (European Centre for Disease Prevention and Control et al., 2015).

The AMC in animals was extracted from the European Surveillance of Veterinary Antimicrobial Consumption Net (ESVAC-Net). It was measured by the overall sales in food-producing animals, including horses by country, and typically reported in milligrams of active substance per kilogram of estimated biomass and per year. Detailed information on the variable definitions and sources are shown in **Table 1**. All variables were transformed into the natural logarithm in the analysis.

**Table 1:** Variable definitions and sources.

Variable name	Definitions	Unit of measure	Sources
*E. coli* resistance rates	*Escherichia coli* resistance rates to fluoroquinolones	%	European Antimicrobial Resistance Surveillance Network (EARS-Net)
*P. aeruginosa* resistance rates	*Pseudomonas aeruginosa* resistance rates to fluoroquinolones		
MS	The number of medical staff	Number per 100,000 population	Eurostat Database
VP	The number of veterinary professionals	Number per 100,000 population	World Organization for Animal Health (OIE)
HAMC	Fluoroquinolone consumption in humans	mg/kg	European Surveillance of Antimicrobial Consumption Net (ESAC-Net)
VAMC	Fluoroquinolone consumption in food-producing animals	mg/kg	European Surveillance of Veterinary Antimicrobial Consumption Net (ESVAC-Net)

### Empirical Analysis

The hypothesis of our study based on the One Health perspective was that the increasing number of healthcare professionals and the decreasing amount of AMC in both human and animal fields may be significant factors of lower AMR rates in the EU. To verify our hypothesis and compare the impacts of factors on AMR rates, the static and dynamic panel data models were employed. We constructed the static model in Equation (1):

(1) $$\begin{array}{l} \ln\;\mathrm{AM}R_{i,t}=\beta_{0}+\beta_{1}\;\ln\;MS_{i,t}\;+\beta_{2}\;\ln\;VP_{i,t}+\beta_{3}\;\ln\;\mathrm{HAM}C_{i,t}\\ \;+\beta_{4}\;ln\;\mathrm{VAM}C_{i,t}+\mu_{i}+\theta_{t}+\varepsilon_{i,t} \end{array}$$

where i denotes country (i = 1, 2,…, 29) and t denotes time (t = 1, 2,…, 12). The disturbance term *µ*_i_ was assumed to be the unobserved country-specific fixed effect, *θ*_t_ was a time-specific intercept, and *ε*_i,*t*_ was random disturbance. The *β* terms were partial correlation coefficients.

This static model can be estimated through a fixed-effects (FE) or random-effects (RE) approach. Panel robust standard errors clustered at the country were used in all analyses of our paper (Bertrand et al., 2004). The RE models assumed that time-invariant variables were uncorrelated with the time-varying covariates, while the FE models allowed these variables to freely correlate (Halaby, 2004). The choice between the RE and FE estimators was based upon the standard Hausman test, whose null hypothesis was that *µ*_i_ was uncorrelated with time-varying variables and the observed time-invariant variables (Gardiner et al., 2009). The FE estimator was preferred if the null hypothesis was rejected (Bramati and Croux, 2007; Bell and Jones, 2014).

The dynamic models introduced a lagged variable as an explanatory variable and introduced instrumental variables by setting moment conditions to handle the autocorrelation of the dependent variables (Blundell and Bond, 1998), which could explore the possible endogeneity of the dependent variables (da Silva and Cerqueira, 2017). The dynamic model used for explaining AMR is shown in Equation (2):

(2) $$\begin{array}{l} \ln\;\mathrm{AM}R_{i,t}=\beta_{0}+\sum_{k=1}^{m}\gamma_{k}\;\ln\;\mathrm{AM}R_{i,t}+\beta_{1}\;\ln\;MS_{i,t}+\beta_{2}\;\ln\;VP_{i,t}\\ \;+\beta_{3}\;\ln\;\mathrm{HAM}C_{i,t}+\;\beta_{4}\;\ln\;\mathrm{VAM}C_{i,t}+\mu_{i}+\theta_{t}+\varepsilon_{i,t} \end{array}$$

where m represents the maximum lag order of the dependent variable. Previous studies had suggested that two lags are sufficient to ensure dynamic completeness (Wintoki et al., 2012). Therefore, we set m = 1 and m = 2 in this study.

Stata 12.0 was used for all the data analysis. The dynamic panel data models were analyzed by the system generalized method of moments (GMM) estimator. This technique consistently estimated the equation even in the presence of endogenous explanatory variables and measurement error and thus allowed us to be more confident in inferring causality (Roodman, 2009). In order to avoid the limitation of weak instrumental variables and the finite-sample bias to a large extent, one-step robust variants of the system GMM estimator were employed (Arellano and Bond, 1991). The applicability of system GMM was examined by Arellano–Bond test and Sargan test. The former test was established on the hypothesis that there is no autocorrelation of disturbance terms, and the latter examines whether all instrument variables are valid. The dynamic model was supposed to be valid if the null hypotheses of these two tests were not rejected.

## Results

### Summary Statistics

**Figure 1:**
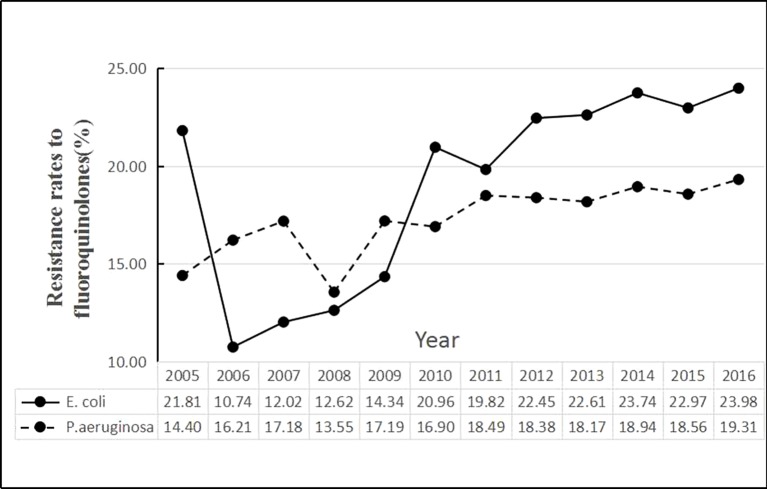
Trends in antimicrobial resistance (AMR) rates among 29 countries from 2005 to 2016.

The time series of average rates of *E. coli* and *P. aeruginosa* in **Figure 1** showed that the *E. coli* resistance rates were higher than *P. aeruginosa* since 2010. During the period, the average rate of *E. coli* was 21.03%, the minimum being 4.68% for Norway and the maximum being 51.85% for Cyprus. The average rate of *P. aeruginosa* was 17.91%, with the maximum and the minimum values being 0 for Iceland and 61.96% for Romania, respectively (**Table 2**).

**Table 2:** Summary statistics of variables.

Variable name	Mean	Std	Min	Max
*E. coli* resistance rates	21.03	10.40	4.68	51.85
*P. aeruginosa* resistance rates	17.91	12.63	0	61.96
MS	1,359.76	412.50	418.22	2,258.34
VP	54.40	25.24	2.44	160.63
HAMC	7.55	4.08	2.23	26.95
VAMC	1.99	2.86	0	11.29

Std refers to standard deviation.

**Figure 2:**
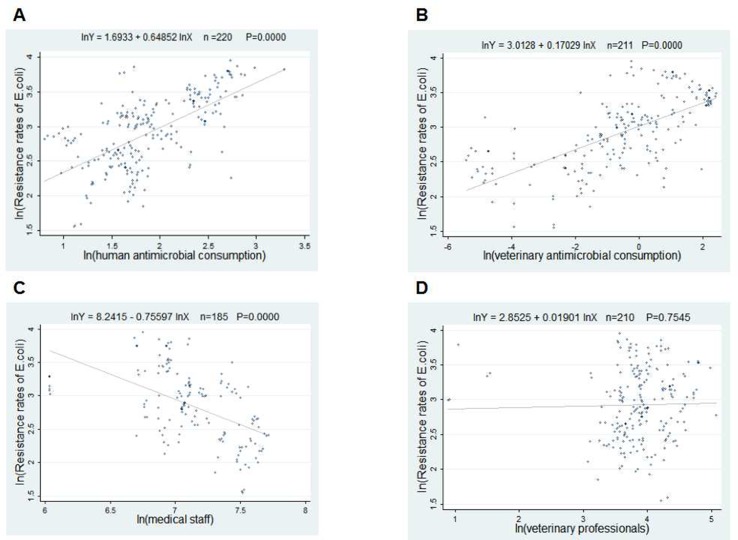
Linear relationships between attributable risk factors and *Escherichia coli* resistance. Notes: The trend line in each plot denotes the fitted line. **(A)** The linear relationship between the human antimicrobial consumption and the *E. coli* resistance rates to FQs (n = 220, P = 0.0000). **(B)** The linear relationship between the veterinary antimicrobial consumption and the *E. coli* resistance rates to FQs (n = 211, P = 0.0000). **(C)** The linear relationship between the medical staff and the *E. coli* resistance rates to FQs (n = 185, P = 0.0000). **(D)** The linear relationship between the veterinary professionals and the *E. coli* resistance rates to FQs (n = 210, P = 0.0000).

**Figure 3:**
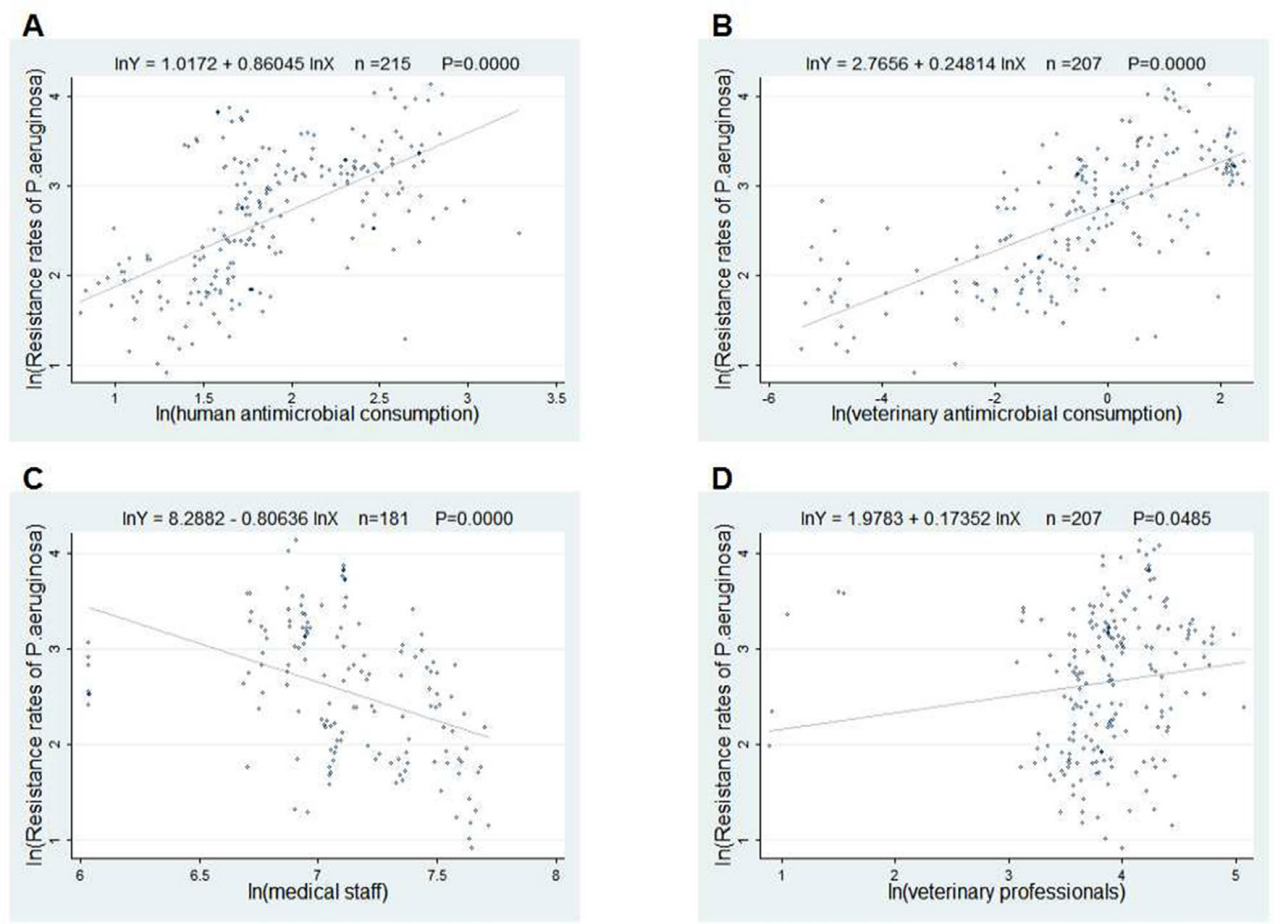
Linear relationships between attributable risk factors and *Pseudomonas aeruginosa* resistance. Notes: The trend line in each plot denotes the fitted line. **(A)** The linear relationship between the human antimicrobial consumption and the *P. aeruginosa* resistance rates to FQs (n = 215, P = 0.0000). **(B)** The linear relationship between the veterinary antimicrobial consumption and the *P. aeruginosa* resistance rates to FQs (n = 207, P = 0.0000). **(C)** The linear relationship between the medical staff and the *P. aeruginosa* resistance rates to FQs (n = 181, P = 0.0000). **(D)** The linear relationship between the veterinary professionals and the *P. aeruginosa* resistance rates to FQs (n = 207, P = 0.0485).

The scatter plots in **Figures 2** and **3** showed the linear relationships between FQ resistance rates and their related factors. The human and veterinary AMC were positively associated with the FQ resistance (all p = 0.0000), and the number of medical staff was negatively correlated with FQ resistance (both p = 0.000), while the positive correlation between veterinary professionals and FQ resistance (P = 0.0485) was only found in **Figure 3**.

### Relationship Between Resistance of *E. Coli* and Its Related Factors

In **Table 3**, based on the Hausman test (Coef. = 12.23, p = 0.016), the FE model was preferred because the null hypothesis was rejected. However, the FE model in column (1) was not statistically significant (F = 2.19, P > 0.05). The dynamic model of one lag in column (3) was supposed to be invalid, as the null hypothesis of the Sargan test was rejected (P = 0.000). The two-lag result of the dynamic model in column (4) displayed that the 10% increase in veterinary professionals was significantly correlated with the 0.57% decrease of *E. coli* resistance rates (Coef. = −0.057, p = 0.030). The coefficient for the first lag of the *E. coli* resistance rates in column (4) indicated a positive tendency of persistence in resistance over time (Coef. = 0.769, p = 0.000).

**Table 3:** Static and dynamic models of related factors to E. coli resistance rates.

Variables	Static		Dynamic	
	FE model (1)	RE model (2)	SYS-GMM (3)	SYS-GMM (4)
			One lag of the dependent variable	Two lags of the dependent variable
lnAMR(t-1)			0.292***	0.769***
			(0.085)	(0.059)
lnAMR(t-2)				0.022
				(0.052)
lnMS	0.008	−0.275	−0.762**	−0.296
	(0.609)	(0.236)	(0.374)	(0.236)
lnVP	−0.098	−0.073	−0.190	−0.057**
	(0.061)	(0.049)	(0.029)	(0.026)
lnHAMC	0.491	0.382**	0.332	0.095
	(0.294)	(0.150)	(0.221)	(0.094)
lnVAMC	−0.090	0.032	0.023	−0.005
	(0.057)	(0.040)	(0.046)	(0.027)
Constant	3.993	4.411**	6.960**	2.783
	(4.285)	(1.710)	(2.829)	(1.852)
No. obs.	131	131	108	88
F	2.19	44.90***		
Hausman test	12.23**			
Wald χ^2^			287.74***	1,025.10***
Arellano–Bond test				
AR (1) (p-value)			[0.147]	[0.011]**
AR (2) (p-value)			[0.171]	[0.354]
Sargan test (p-value)			[0.000]***	[0.849]

FE model = fixed-effect model; RE model = random-effect model; SYS- GMM = system generalized method of moments; AMR = antimicrobial resistance; numbers inside () are robust standard errors; numbers inside [] are p-values; AR (1) = autocorrelation test of order 1; AR (2) = autocorrelation test of order 2; *, **, and *** represent, respectively, 10%, 5%, and 1% significance levels.

### Relationship Between Resistance of *P. Aeruginosa* and Its Related Factors

Similarly, in **Table 4**, the FE model was preferred based on the Hausman test (Coef. = 23.01, p = 0.000). The FE model in column (1) showed that a 10% increase of medical staff was associated with 32.44% decline of *P. aeruginosa* resistance rates (Coef. = −3.244, p = 0.000). And the 10% increase in the human and veterinary AMCs were related to a 10.06% increase and 1.65% decrease of *P. aeruginosa* resistance rates, respectively (Coef. = 1.006, −0.165; p = 0.006, 0.019, respectively). In the dynamic models, the results in column (3) denote that 10% human AMC was positively correlated with an 8.04% increase of *P. aeruginosa* resistance rates (Coef. = 0.804, p = 0.001). The dynamic model of two lags in column (4) was supposed to be invalid, as the null hypothesis of the Sargan test was rejected (P = 0.088).

**Table 4:** Static and dynamic models of related factors to *P. aeruginosa* resistance rates.

Variables	Static		Dynamic	
	FE model (1)	RE model (2)	SYS-GMM (3)	SYS-GMM (4)
			One lag of the dependent variable	Two lags of the dependent variable
lnAMR(t-1)			0.136	0.189
			(0.199)	(0.221)
lnAMR(t-2)				0.108
				(0.075)
lnMS	−3.244***	−0.394	−0.439	−0.549
	(0.784)	(0.449)	(0.651)	(0.693)
lnVP	−0.001	0.020	0.037	−0.036
	(0.061)	(0.070)	(0.045)	(0.040)
lnHAMC	1.006***	0.478**	0.804***	0.754***
	(0.331)	(0.211)	(0.252)	(0.253)
lnVAMC	−0.165**	0.050	0.085	0.050
	(0.065)	(0.049)	(0.148)	(0.149)
Constant	23.752***	4.420	4.072	4.522
	(5.629)	(3.192)	(4.207)	(4.451)
No. obs.	130	130	105	84
F	7.84***	25.08***		
Hausman test	23.01***			
Wald χ^2^			90.63***	146.59***
Arellano–Bond test				
AP (1) (p-value)			[0.013]**	[0.342]
AP (2) (p-value)			[0.421]	[0.322]
Sargan test (p-value)			[0.195]	[0.088]*

*, **, and *** represent, respectively, 10%, 5%, and 1% significance levels.

## Discussion

The panel data set allows us to further explain related factors to FQ resistance rates in the EU from the One Health perspective; both human and veterinary factors were included in each analytical model. And several points are worth discussing.

### Healthcare Staff in Both Human and Animal Fields Are Closely Associated With FQ Resistance

The results indicate that the increasing number of medical staff is a significant factor of lower *P. aeruginosa* resistance rates to FQs. This One Health perspective finding is consistent with prior studies, which have exemplified that the AMR rates are significantly associated with the multi-disciplinary antimicrobial stewardship teams (ASPs) (Ansari et al., 2003; Carling et al., 2003). For example, Wu et al. found that the extensive implementation of the ASPs by specialized staff was effective in reducing AMR of Gram-positive bacteria (p = 0.013) and Gram-negative bacteria (p < 0.001), and predominant species included *E. coli* and *P. aeruginosa* (both p < 0.05) (Wu et al., 2017). Compared with experts in animal husbandry or agriculture, medical staff have been recommended and required more for minimizing AMR (Pflomm, 2002). Their knowledge, attitude, and prescribing habits could determine antimicrobial use, which eventually influences AMR rates. Our study provides the ecological evidence to confirm that the number of medical staff could be an important factor of FQ resistance.

The increase of veterinary staff was correlated with a limited but significant decline of *E. coli* resistance rates to FQs. That is, a large increase of veterinary professionals is associated with a little decrease of FQ resistance. Present studies have explored the relationship between veterinary professionals and AMR in the animal field (Sadiq et al., 2018). These studies found that veterinarians’ knowledge, beliefs, and practices played important role in combating AMR in animals (Cattaneo et al., 2009). This finding highlights the significant relationship between the number of veterinary professionals and FQ resistance. As veterinarians are considered as highly influential referents combating AMR, there might be a huge role for veterinarians in motivating and advising farmers to take preventive control measures (Ellis-Iversen et al., 2010). Moreover, One Health AMR inter-agency ASPs have been described and advocated by many EU countries to control the development of AMR. The local One Health AMR groups in Sweden made great contributions to a reduction in AMC and lowered AMR rates over 10 years, without measurable negative consequences (Moelstad et al., 2008). Hence, our findings implicate that the closer medico-veterinary collaboration based on One Health approach may be needed to effectively control the development of AMR or to create comprehensive guidelines to promote prudent use and careful restriction of antimicrobial drugs.

### The Relationships Between AMC in Human and Animal Fields and FQ Resistance are Mixed

Additionally, the increase of human AMC is also an important influencing factor associated with higher FQ resistance. The results presented in this paper are in line with those presented in several other papers (Mutnick et al., 2004; Dalhoff, 2012). For instance, high correlations were found between the use of meropenem (r = 0.98), ciprofloxacin (r = 0.92), and ceftazidime (r = 0.83) and the resistance of *P. aeruginosa* to these agents by linear regression analysis over a period of 3 years in 10–15 medical centers (Mutnick et al., 2004). In contrast, Livermore et al. noticed that the rates of FQ resistance among *E. coli* isolates increased, despite the decline in the rates of prescription of FQs in the community (Livermore et al., 2002). In addition, an association between consumption and resistance may not be found if there is a delay between the reduction of AMC and the subsequent decrease of AMR (Bergman et al., 2009). In recent years, a few studies have started exploring the relationship between AMC and AMR through a macro perspective, which overcomes details of the behavior of individual units. The EU second joint report showed a 1 mg/kg increase in the human consumption of FQs resulted an increase of the risk of invasive *E. coli* resistance to FQs of around 56%, 48%, and 53% for the years 2013, 2014, and 2015, respectively (OR = 1.56, 1.48, 1.53) (European Centre for Disease Prevention and Control et al., 2017). The results of our panel data analysis, which could go beyond the effect of delay and individual behavior, provide reliable evidence to support the positive association between human AMC and FQ resistance.

However, the result that the decrease of AMC in food-producing animals was associated with the increase of *P. aeruginosa* resistance rates was contrary to our hypothesis. We speculate that this result may be influenced by the increasing proportion of therapeutic antibiotic use in total AMC and the food trade worldwide. Evidence showed that the ban of antimicrobial growth promoters (AGPs) reduced the food animal reservoir of AMR. And total veterinary AMC in Denmark dropped by more than 50% from 1992 to 2008 after the termination of use of AGPs was implemented (Levy, 2014). Nevertheless, increased therapeutic use of antimicrobials and meat imports make contributions to continuing resistance (Aarestrup et al., 2001; Alban et al., 2008). In low- and middle-income countries, some production systems still use antimicrobials to keep animals healthy and maintain productivity for an unprecedented growth in demand for animal protein (Tilman et al., 2011). Besides, it is worth noting that food animals and foods of animal origin are traded worldwide. Rasmussen et al. revealed that *Campylobacter jejuni* resistance to ciprofloxacin, nalidixic acid, and tetracycline was significantly higher in imported chicken meat compared to Danish broiler meat in years during the 2002-through-2007 period (Skjot-Rasmussen et al., 2009). Therefore, the resistant genes in imported food animals may also bring an influence on local AMR. Better surveillance of international trade and movement of these animals and animal products may be required so that their impact on the development of AMR could be more accurately assessed (Queenan et al., 2016). On the other hand, from a methodological perspective, such findings may be influenced by the model specification, as statistical significance is only found in the static model, and more studies should be encouraged to further explain the relationship when more detailed data are obtained.

## Conclusions

The professional resources and AMC in both human and animal fields are the significant factors for AMR rates from the One Health perspective based on the main results. The increase of medical and veterinary staff is closely associated with a decrease of AMR rates. The increase of human AMC is associated with an increase of *P. aeruginosa* resistance rates, but the increase of veterinary AMC is correlated with a decrease of *P. aeruginosa* resistance rates to FQs. Several limitations of our study and models deserve mentioning. Firstly, food animals are not the only reservoir of antibiotic-resistant bacteria; pets, birds, insects, wild rodents, and other animals should be included if they are available. Secondly, the present study does not consider environmental factors, behavioral factors, and other determinants of AMR. Thirdly, regardless of other antimicrobials, our study only included the FQ resistance rates due to the data availability. Hence, more studies should be undertaken to provide further evidence and guidance on the relationship between AMR and these main factors if more data are available.

## Data Availability Statement

Publicly available datasets were analyzed in this study. These data can be found here: European Antimicrobial Resistance Surveillance Network (EARS-Net), Eurostat Database, World Organization for Animal Health (OIE), European Surveillance of Antimicrobial Consumption Net (ESAC-Net), and European Surveillance of Veterinary Antimicrobial Consumption Net (ESVAC-Net).

## Author Contributions

DZ was involved in statistical analysis, data interpretation, and the manuscript draft. XZ conducted the study design, data interpretation, and manuscript writing during the whole process. YC made significant contributions to the data interpretation. All authors read and approved the final manuscript.

## Funding

This work was supported by the National Natural Science Foundation of China (71473098) and the 2017 Independent Innovation Fund—Major and Cross-Project (5003516009). The funders played no role in study design, data collection and analysis, decision to publish, or manuscript preparation.

## Conflict of Interest

The authors declare that the research was conducted in the absence of any commercial or financial relationships that could be construed as a potential conflict of interest.
