# Supplementary material for: Estimating Factors Related to Fluoroquinolone Resistance Based on One Health Perspective: Static and Dynamic Panel Data Analyses From Europe
Source: Front Pharmacol. 2019 Oct 3;10:1145. doi: 10.3389/fphar.2019.01145 (PMC6787557; doi:10.3389/fphar.2019.01145)
Supplement: Supplementary file 1 [file Table_1.doc]

Appendix A. Description of data availability in 29 countries

| Country | E. coli resistance rates | P. aeruginosa  resistance rates | MS | VP | HAMC | VAMC |
| --- | --- | --- | --- | --- | --- | --- |
| Austria | 2010-2016 | 2010-2016 | 2005-2015 | 2005-2016 | 2010-2016 | 2010-2016 |
| Belgium | 2010-2016 | 2010-2016 | 2005-2011 | 2005-2016 | 2010-2016 | 2010-2016 |
| Bulgaria | 2011-2016 | 2011-2016 | 2005-2010 | 2005-2016 | 2011-2016 | 2011-2016 |
| Cyprus | 2011-2016 | 2011-2016 | 2005-2015 | 2005-2016 | 2011-2016 | 2011-2016 |
| Czech Republic | 2005-2015 | 2005-2015 | 2005-2013 | 2005-2016 | 2005-2015 | 2010-2015 |
| Germany | 2011-2016 | 2011-2016 | 2005-2015 | 2005-2016 | 2011-2016 | 2011-2016 |
| Denmark | 2005-2016 | 2005-2016 | 2006-2015 | 2008-2016 | 2005-2016 | 2005-2016 |
| Estonia | 2010-2016 | 2010-2016 | 2005-2015 | 2005-2015 | 2010-2016 | 2010-2016 |
| Greece | 2015-2016 | 2015-2016 | 2005-2015 | 2005-2015 | 2015-2016 | 2015-2016 |
| Spain | 2010-2016 | 2010-2016 | 2005-2015 | 2005-2016 | 2010-2016 | 2010-2016 |
| Finland | 2005-2016 | 2005-2016 | 2005-2011 | 2005-2016 | 2005-2016 | 2005-2016 |
| France | 2005-2016 | 2005-2016 | 2005-2016 | 2005-2016 | 2005-2016 | 2005-2016 |
| Croatia | 2005-2016 | 2005-2016 | 2005-2016 | 2005-2016 | 2005-2016 | 2014-2016 |
| Hungary | 2014-2016 | 2014-2016 | 2005-2016 | 2005-2016 | 2014-2016 | 2010-2016 |
| Ireland | 2005-2016 | 2005-2016 | 2005-2013 | 2005-2016 | 2005-2016 | 2010-2016 |
| Iceland | 2010-2016 | 2010-2016 | 2005-2016 | 2005-2016 | 2010-2016 | 2010-2016 |
| Italy | 2011-2016 | 2011-2016 | 2005-2016 | 2005-2016 | 2011-2016 | 2011-2016 |
| Lithuania | 2010-2016 | 2010-2016 | 2005-2015 | 2005-2016 | 2010-2016 | 2010-2016 |
| Luxembourg | 2012-2016 | 2012-2016 | 2005-2013 | 2005-2013 | 2012-2016 | 2012-2016 |
| Latvia | 2010-2016 | 2010-2016 | 2005-2015 | 2005-2016 | 2010-2016 | 2010-2016 |
| Netherlands | 2005-2016 | 2005-2016 | 2005-2015 | 2005-2016 | 2005-2016 | 2005-2016 |
| Norway | 2005-2016 | 2005-2016 | 2005-2015 | 2005-2016 | 2005-2016 | 2005-2016 |
| Poland | 2011-2016 | 2011-2016 | 2005-2015 | 2005-2016 | 2011-2016 | 2011-2016 |
| Portugal | 2010-2016 | 2010-2016 | 2005-2015 | 2005-2016 | 2010-2016 | 2010-2016 |
| Romania | 2014-2016 | 2014-2016 | 2005-2016 | 2005-2016 | 2014-2016 | 2014-2016 |
| Sweden | 2005-2016 | 2005-2016 | 2005-2014 | 2007-2016 | 2005-2016 | 2005-2016 |
| Slovenia | 2010-2016 | 2010-2016 | 2005-2014 | 2005-2016 | 2010-2016 | 2010-2016 |
| Slovakia | 2011-2016 | 2011-2016 | 2005-2014 | 2005-2016 | 2011-2016 | 2011-2016 |
| United Kingdom | 2005-2016 | 2005-2016 | 2005-2016 | 2005-2016 | 2005-2016 | 2005-2016 |
